# Supplementary material for: Visualization of peripheral nerves in developing and regenerating limbs using a novel peripherin reporter line of Xenopus laevis
Source: Biol Open. 2026 Jun 10;15(6):bio062651. doi: 10.1242/bio.062651 (PMC13312924; doi:10.1242/bio.062651)
Supplement: Supplementary information [file biolopen-15-062651-s1.pdf]

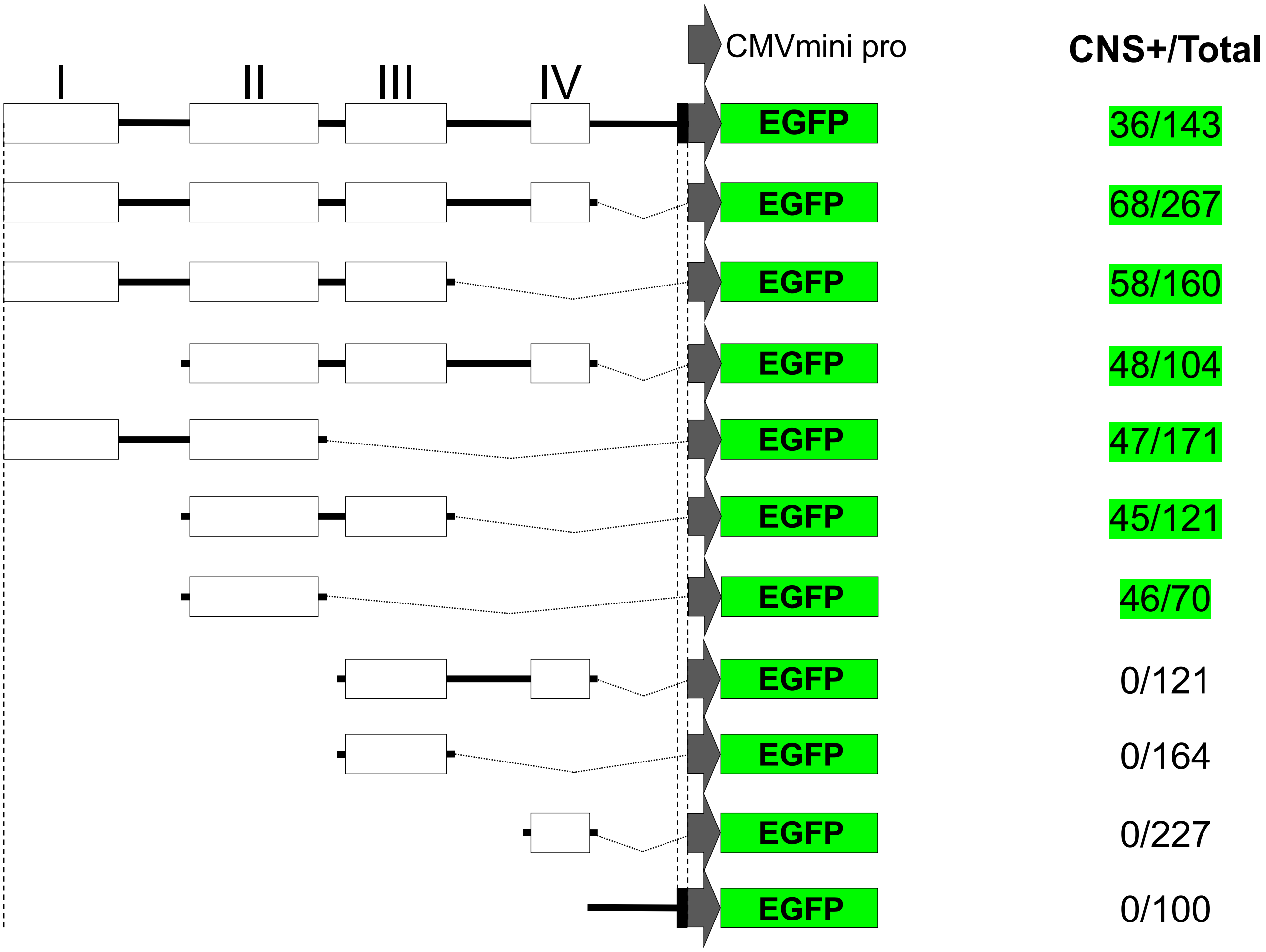

Fig. S1. Deletion and enhancer analyses of intron 1 of the *prph* gene in transgenic *Xenopus* Data from sequential deletion analyses of the *prph* intron 1 sequence are summarized in Figure 2A. The number of CNS-specific EGFP expressing transgenic embryos at the tailbud stage (EGFP positive embryos/total embryos) is indicated for each construct. White boxes, a black box, and a gray arrow represent homology regions, exon, and the CMV minimal promoter, respectively.

Biology Open • Supplementary information

Biology Open (2026): doi:10.1242/bio.062651: Supplementary information

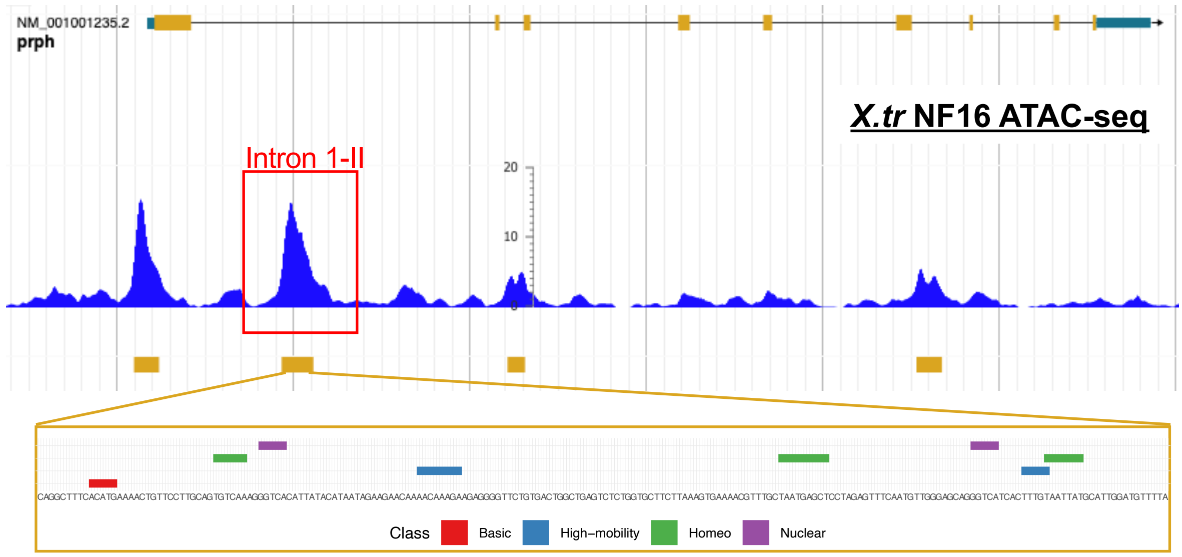

motif	TF	score	motif	TF	score
	POU6F2	14.799452		SOX10	11.610084
	POU6F1	12.783767		DLX5	11.605389
	SOX4	12.535857		HOXA3	11.563704
	HOXB7	12.223566		MEOX1	11.529902
	LHX5	12.081846		HOXA2	11.304736
	HOXB1	11.695127		EMX1	11.233512
	MXI1	11.675601		EMX2	11.207822

Fig. S2. *Cis*-element analysis of Homology Region II (Intron 1-II) of the *X. tropicalis prph* gene ATAC-seq peaks at the neurula stage are shown with the corresponding sequences. Predicted transcription factor binding motifs are highlighted with colored boxes. ATAC-seq data are from Xenbase. *Cis*-elements of Region II in intron 1 are predicted by JASPAR. Abbreviations: Basic, Basic helix-loop-helix type transcription factors; High-mobility, High-mobility group domain type transcription factors; Homeo, Homeo domain type transcription factors; Nuclear, Nuclear receptors with C4 zinc finger type transcription factors.

Biology Open • Supplementary information

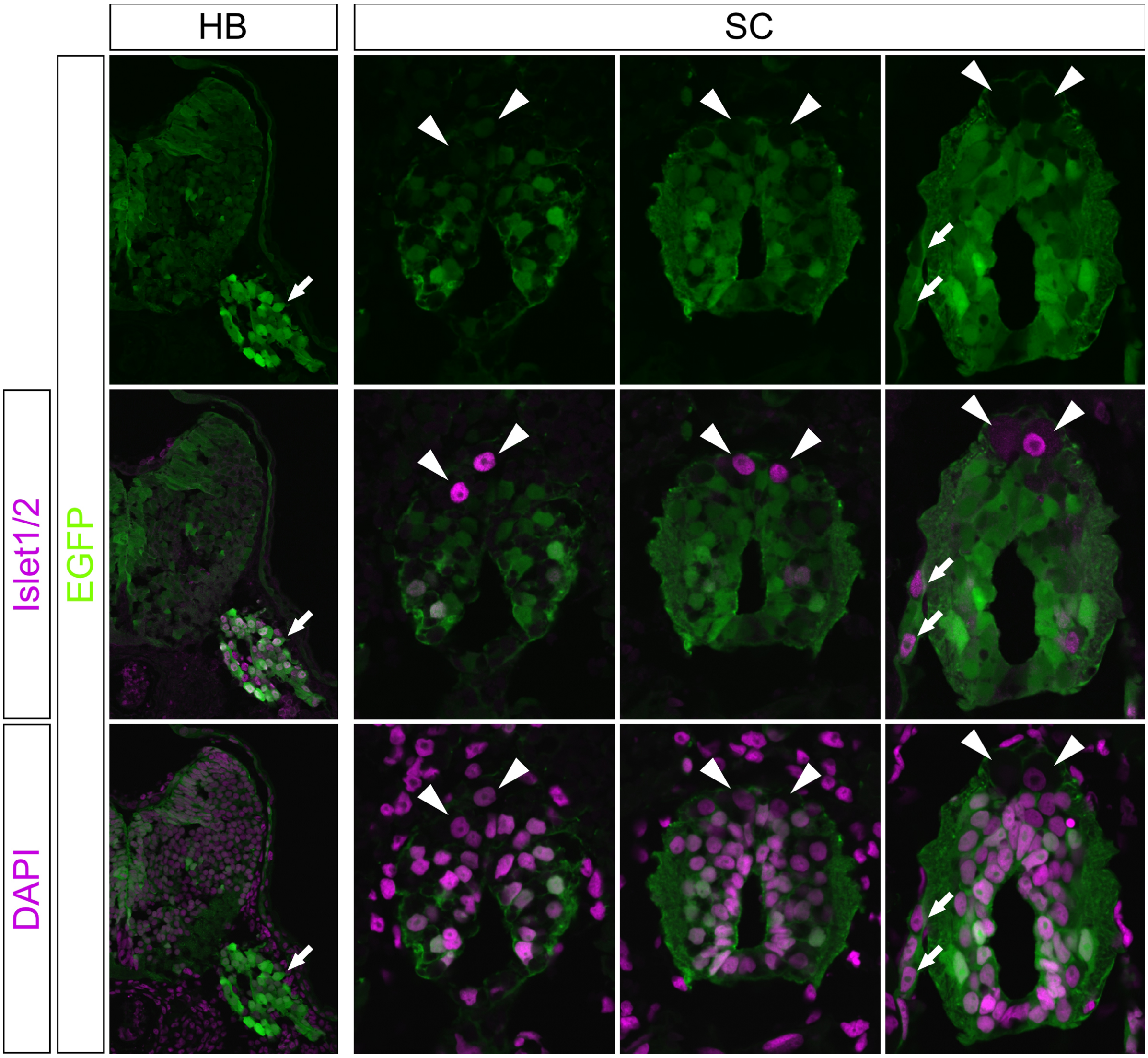

Fig. S3. Immunohistochemical analysis of EGFP reporter in the *prph* transgenic embryos. Transverse sections of stage 42 *prph*:EGFP embryos at hindbrain (HB) and spinal cord (SC) levels were immunostained for EGFP (green) and Islet1/2 (magenta in middle panels) or DAPI (magenta in lower panels). In the hindbrain, arrows indicate EGFP-positive trigeminal ganglion neurons. In the spinal cord, arrows and arrowheads indicate EGFP-positive dorsal root ganglia (DRGs) and EGFP-negative Rohon-Beard (RB) cells, respectively. EGFP expression is prominent in ventral neurons but absent in dorsal RB cells.

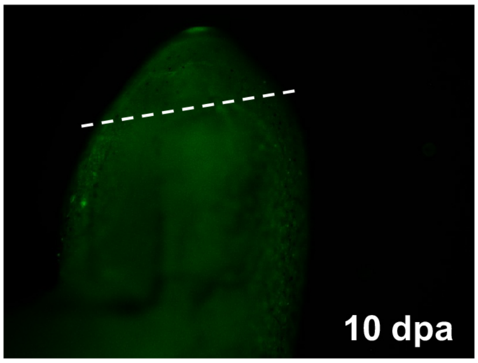

Fig. S4. No fluorescence in the wild-type froglets following amputation. A dotted line indicates the amputation plane. Neither auto- nor nerve-specific fluorescence was observed at 10 dpa (n=3).

Biology Open • Supplementary information

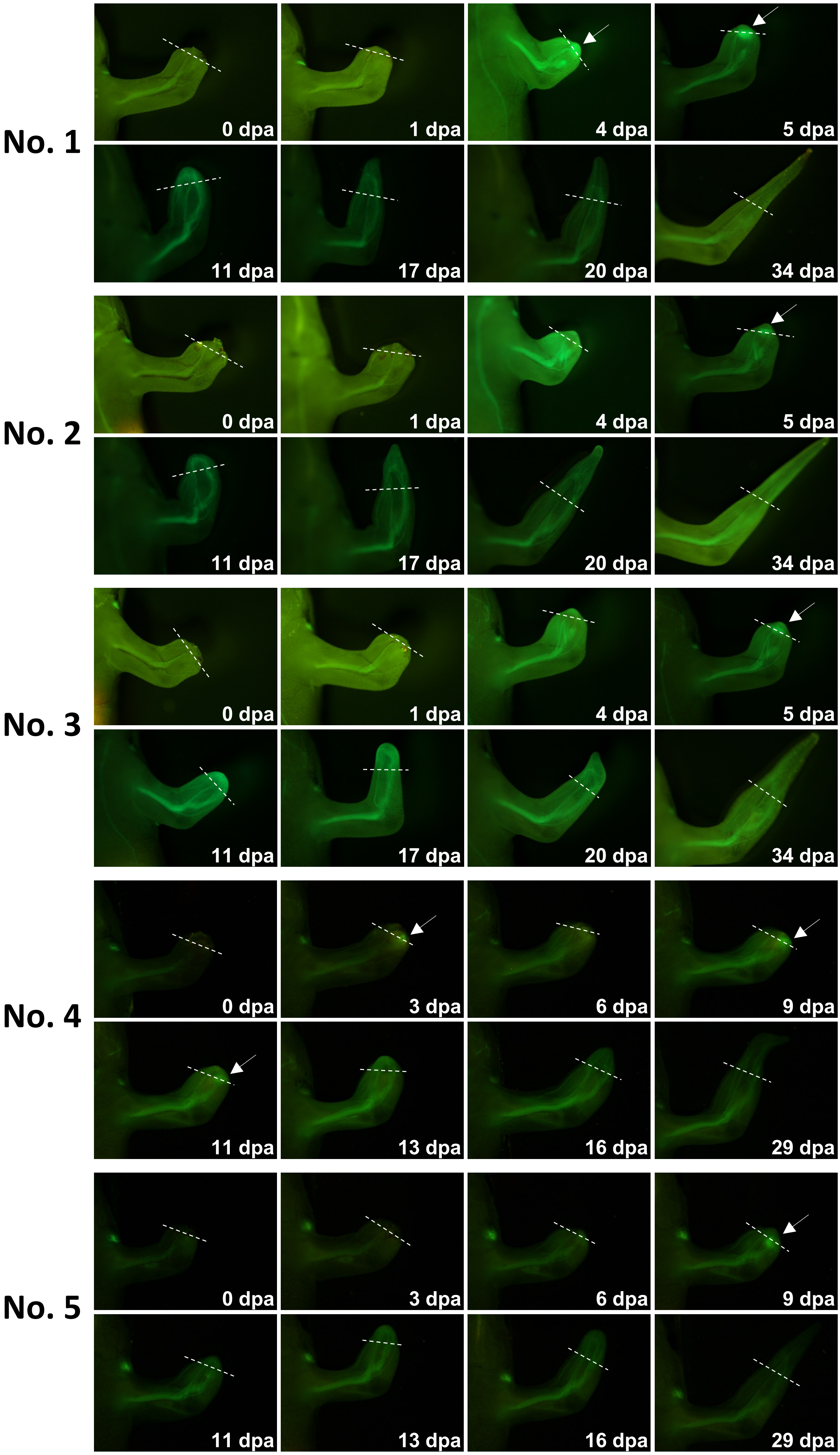
No. 1
0 dpa
1 dpa
4 dpa
5 dpa
11 dpa
17 dpa
20 dpa
34 dpa
No. 2
0 dpa
1 dpa
4 dpa
5 dpa
11 dpa
17 dpa
20 dpa
34 dpa
No. 3
0 dpa
1 dpa
4 dpa
5 dpa
11 dpa
17 dpa
20 dpa
34 dpa
No. 4
0 dpa
3 dpa
6 dpa
9 dpa
11 dpa
13 dpa
16 dpa
29 dpa
No. 5
0 dpa
3 dpa
6 dpa
9 dpa
11 dpa
13 dpa
16 dpa
29 dpa

Fig. S5. Time-course imaging of EGFP reporter fluorescence in the *prph* transgenic froglets following amputation. Dotted lines indicate the amputation plane. Arrows denote transient and ectopic EGFP expression. These ectopic signals appeared 4–9 dpa, then diminished and became undetectable by 13 dpa (total n= 6/6 including Figure 5A).

Biology Open • Supplementary information

<u>Sequence of 914 bp *prph.L* promoter</u>

gtcgacggatcgcggccgcgaattctcgatcatctctgcagggggctaggggaataacaattaggggagtcaaccctgcaaatactggtggtctaggagaaggggagctgccatgtgca
atttatgtgagattatttatttaaaaaaacttgatttaatagggtcgtgaaaaaactcgataaaatcaagctaaaacaaattgtacaattctgaaaaaagtcagattttccggctaaacc
cagcgcagaccacgataacttgaaattaggatagctgcctctcccattgacaggtctgagatggcagatttttggattctatctttttgcagaattgagtataataaatctcgaaaaaaa
tctagtttttttttcctctaaaaattagagttttctagtgataaaaaaatgcaaatattaagagatttaacaactcgtgctttaataaataaccccccaacatctctggtgttatggggcc
ctaaaaatattttgctgtgcggtcccttcattttagttacaacattaaaggggatggacaggggacatctaaatcctatattcatgtgaatataaatatattgttagttgtttctgagct
gtgttccattcacttacagccacagagcataagccgttgaatagaaggatgaaagtaaatctgagtgctgtaggaagatgcacagagtgagttcagcatataatgggtgggtgtggtgga
tgcagatcccccctcctccctctgctcagggttatgcatctatgtggttccacagggaggggctgagtctgcacaataagatgctgcatgctggcttataaagcagcctgccatactctca
gactttcttctcaacatcactcgagaaCTAAGTACTAACATCCAGCTTAAGTGCTTGGGAATTCCTTGCTCAT

Blue, 5´UTR

Biology Open • Supplementary information

Sequence of *prph.L* Intron I (4,718 bp)

```
gtaaggtgccaggggtttagaggcagatggaggaatgtagttatttctgaggggttataagtatacaaacatgtgaagaacttctaagaagtctgaaacatttcattggtgatggggt
              P1F ->
ggccatatagatatacaaaagagaagaaactgaccagtgcatattgcctttccccatattttatttgtaattaaatttttgcatatattttcaaaagcagttttaggtaaagttcatata
gtaccccctattaacctctggttgtatgtttataggctggtattccttgttaggtggcatttcgtgggaaaagtgtctcctggccaaagtattggtcacacagactgattctccctgactg
tagaatttatatgggagagatctcaagaaccaggcctatttctgaaaacataagacccctccatgggtgggtgtctgttatgtgtcatgtggtggcattagattagatgcgggcagtcta
gacataggcagggtgtggccatgccactaaatattatgctctgtatcttctgtggcttactttgctacttctactgttcatcctggaacaatgctgataaagggaatgtactgaaggttt
taacaggcaacttttttataatacaaaatattactgccaaagaaataactgttacactatgccatgtaactccttgttaacatgtattttatttgtatataatacacaaaagtcatgaata
                                                                                                                  <- P1R
tcttgtaaattatatccttataaacggtgagtagtgatgtcatcagttataaacggtgagtagtgatgtcatttctgtcacatgactcactaaaatttgtgtattataataaagtacccc
cagttgtaaaatatgaggatattataagttacctcggagttccatgaccatataaaagcacgaggcctcgtgtttttatatggtcatgaaactcctcggtaacttataatatccttatat
tttacaagaggggggtactttattcactatacaacaaatgatccagtccagtcttgttttcttataacagtggcggaagagggaaatacttataatgaagagatggtatcaacaacagccc
ttgctgcattactattctcagcttaaaataagtaatgccacatgttatagttcagcaacaaaaagtacttgttctattacaatgtatatccatgtgataatgaacaaaaacgataaattc
                                            P2F ->
agaaggttgatttcattattccaatcagactagactggttagttctagcttcaaattagttgtcaagggttacaggatcaaggcaaactttatggccgtttgtgcaatacgcccttgctt
tcccaggctttcacatgaaaactgttccttgcagtgtcaaagggtcacattatacataatagaagaacaaaacaaagaagaggggttctgtgactggctgagtctctggtgcttcttaaa
gtgaaaacgtttgctaatgagctcctagagtttcaatgttgggagcagggtcatcactttgtaattatgcattggatgttttattttttactaacaaatgcacagaaagccagtttcctc
tgtgacaatgtggttgcagttgcaccaatgtgtgatctcattgatcctttgctaatcattggttaatccttcccccctagtgctcttatcttacataggtcattgcacagctggccactct
cttacccactccctatgggcaagattaaaggttaggggaagagtgcagcaccagtatgggcagaagttgaaaagcacagatgtatggataatggcaaaaagaacaatgaataaaggtggg
                                                                                                                       <- P2R
aatgggaatacaggccgtgagcactgcatcaatgagctgatcctcattttaacggaatttttaaacttgtccgattaatatctggctgattttcggccagttattaatcaggtagaccca
tcagcgggccctatagacaggcaggtaagctgttgactctgagcctgagtccaaatctgcctgtgatttcaggaatgctaatcacactgtgttactactaggaaatgaaacattacaatc
                         P3F ->
ccttagtttttcagagcatcattttgtttcaaaattcataattggacagtaccataatcctttaggctgctgccatacaggtcatattcccccctgccattatttagccatctgagtaac
acccaagctgcctgttattggctttaattttggccccaaaagacagatgtaagcattttaggctacatttagtgacaggtgtttttcactatctccagtaacaggtggcacacccctgt
gccatcattctgaagtgcaactaacttctgtttcttttcactttggtataatggaaattataggttgttggctccataaatgtttttgttaaaggcctttaaaaatagtatgtattgtat
tcacatttatagtaaaatgtcaaatagttccatgtggaaaaaaagaagaataacttatatttagagaattgtcatttaggagattgggtgtgtgagcatagacacttattgttctggtac
gcaagactgggtgtgtctatgggggcaggatgaatggtaaaatatctgcaacagggctggcacaatctgcccaagatgatattgagtgttcatatgagtgttggtttatgtatagataac
tggataaacgtgtccaagtcgcaaataattactctaggctgctgaagcatttataagaaacaaatgctttggaaacaacttttactggccaaactagtgctgttcctcttgatgccactg
   <- P3R
ttgtttgcagggataaaaaggtaggtagaaaggttagcatctgtgacagagagtgtttatggactgtctggtggctgatagatgcagtcatccaaatcaggcttagtcagataacattca
tggtggggcagtgtacggccatcttaactgtctgattccagcttgagacaacagattgcaatacaacagaaagtgtgattcagtggtgtaactattcagagccgtccctccctagcaaaa
aacaaatctttaggggtgtcccctacctgcctgcatacccctgctcctgcctacaggtaggagagcagcaaaagagggggggatttctttgcaactatagaggacgcagaccctgtggtctg
cgcccttatatctagttctacttatatccagactgtgttagaaaaagtcaaacctattcatggaaaataattattcaacatcttttacaatatggttgcatgtctacagcaattgctgtt
                                  P4F ->
ccacttaatccacatatacaatacacccttggggatttatagagctcagaatatgagcaatactaaaatctatgggcaggttgtgaaaaaacccataacttctatacattctgttctctg
cgtactctactaaaactgtacatacaacacagtccaacattatgacttgtccactccagataaactttgtagctgattgccccatttaaggggctaaagtgactgcttgccagatatctt
gccaggaattggctagatatctctcggggaggcgggggtagttaaacatttgaatgctgcattgggctactgatttggtcctttgctaccagatcaccataggtctctgttatggtctg
                              <- P4R                   P5F ->
cacgttgtattggaggctaaacaatcagatcagcttgactcagggtgactgaatcaagcaaagagtcacttgtttgtctaacttgcttaaaggaaaactatacccccaaaatgaatactt
aagcaacagatagtttatatcaaattgaatgacatattaaagaatcttaccaaactggaatatatatttacataaatattgcccttttacatctcttgccttgaaccaccatttcgtgac
tctatctgtgctgcctcagagatcacctgaccagaaatactacaacactaactgtaacaggaagaagtgaggaagcaaaaggcagaactctgtctgttaattggctcatgtgaccttaca
tgtggtttgtatgtgtgcacagtgaatcttacgatcccaggggggcggcccttatttttaaaatggcaattttctatttatgattacccaatggcacatactactaaaaaagtatattat
tatgaaaatggttcatttacatgaagcagggttttacacatgagctgttttactcagtatcttttaatagagacctacattgtttgggggtatagttttcctttaataagataatctta
aaacaggtaaatgaccttccttttttgcttcttttaattcatattaaaaaaggtgatgcaaagctaattaaatgaccttcctttttgcttctgtaattcaaagcctcaaaacaacctcaga
tgaacgtgaaattggttagagtgcctttctaaaaacctttgcaatttacattatttttcaagatcttgacagtggctggactgtacaaatggtttaaatgggggtacatagctgaaatga
ctgtacaagaaatgggatgacatggctgaactggagtggtgtgcaaaatatattccaaatttacttctaccatattgagatttggaatgcagtgtagcctgaagaaagtggagagggaag
gttaatgccttattttatcgattacataaaaagagctcacaagtggtaatctttagacttggacaccttatggtatttgttaccatggcttttagtgattttgtttcttttattcctgg
ccagGCTAGATGAAGAAGTTCACAAACGGGAAGATGCAGAGAATAATCTAGTCCTGTTTAGAAAGgtgagtcaaaaaaaaagagtaagaaacacctctgtctgatccagtcaggttaccat
       <- Ex2R
```

Primer sequences are underlined.

Biology Open • Supplementary information

Primers for subcloning of the first intron fragments

Fragment	Forward primers	Reverse primer
I II III IV	P1F	P4R
I II III	P1F	P3R
II III IV	P2F	P4R
I II	P1F	P2R
II III	P2F	P3R
III IV	P3F	P4R
II	P2F	P2R
III	P3F	P3R
IV	P4F	P4R
V	P5F	Ex2R

	Primer list	
Name	sequence	Tm
peripherinG1-exon2-Rv (Ex2R)	5-TGC ATC TTC CCG TTT GTG AAC TTC TTC ATC-3	66
PRPHint1P1-Fw-Hind3 (P1F)	5-GCG AAG CTT AAG GTG CCA GGG GGT TTA GAG-3	69
PRPHint1P2-Fw-Hind3 (P2F)	5-GCG AAG CTT GCC ACA GGT TAA AGT TCA GCA-3	69
PRPHint1P3-Fw-Hind3 (P3F)	5-GCG AAG CTT GGC AGG TAA GCT GTT GAC TCT-3	68
PRPHint1P4-Fw-Hind3 (P4F)	5-GCG AAG CTT CCA GAC TGT GTT AGA AAA AGT-3	62
PRPHint1P5-Fw-Hind3 (P5F)	5-GCG AAG CTT GAA TGC TGC ATT GGG CTA CTG-3	71
PRPHint1P2-Rv-Sal1 (P2R)	5-GCG CGT CGA CCC ATT CCC ACC TTT ATT CAT-3	72
PRPHint1P3-Rv-Sal1 (P3R)	5-GCG CGT CGA CTT GGA CAC GTC TAT CCA GTT-3	70
PRPHint1P4-Rv-Sal1 (P4R)	5-GCG CGT CGA CGT TTA ACT ACC CCC CGC CTC-3	75

Partial sequence of pEGFP(-P)

```
                                          45 EcoRI   55 SalI
        10 Eco47III                 38 HindIII  50 PstI    61 KpnI         76 BamHI
TAGTTATTTAGCGCTACCGGACTCAGATCTCGAGCTCAAGCTTCGAATTCTGCAGTCGACGGTACCGCGGGCCCGGGATCCACCGGCCGGTCGCCACCATGGTGAGCAAGGGCGAGGAGC
TGTTCACCGGGGTGGTGCCCATCCTGGTCGAGCTGGACGGCGACGTAAACGGCCACAAGTTCAGCGTGTCCGGCGAGGGCGAGGGCGATGCCACCTACGGCAAGCTGACCCTGAAGTTCA
TCTGCACCACCGGCAAGCTGCCCGTGCCCTGGCCCACCCTCGTGACCACCCTGACCTACGGCGTGCAGTGCTTCAGCCGCTACCCCGACCACATGAAGCAGCACGACTTCTTCAAGTCCG
CCATGCCCGAAGGCTACGTCCAGGAGCGCACCATCTTCTTCAAGGACGACGGCAACTACAAGACCCGCGCCGAGGTGAAGTTCGAGGGCGACACCCTGGTGAACCGCATCGAGCTGAAGG
GCATCGACTTCAAGGAGGACGGCAACATCCTGGGGCACAAGCTGGAGTACAACTACAACAGCCACAACGTCTATATCATGGCCGACAAGCAGAAGAACGGCATCAAGGTGAACTTCAAGA
TCCGCCACAACATCGAGGACGGCAGCGTGCAGCTCGCCGACCACTACCAGCAGAACACCCCCATCGGCGACGGCCCCGTGCTGCTGCCCGACAACCACTACCTGAGCACCCAGTCCGCCC
TGAGCAAAGACCCCAACGAGAAGCGCGATCACATGGTCCTGCTGGAGTTCGTGACCGCCGCCGGGATCACTCTCGGCATGGACGAGCTGTACAAGTAAAGCGGCCGCGACTCTAGATCAT
```

Green, EGFP coding sequence

Partial sequence of pminCMV-EGFP

```
                                                    56 SalI
        11 Eco47III                 39 HindIII  51 PstI    62 KpnI
TAGTTATTCTAGCGCTACCGGACTCAGATCTCGAGCTCAAGCTTCGAATTCTGCAGTCGACGGTACCGCGGGCCCGGGTCGAGGTAGGCGTGTACGGTGGGAGGCCTATATAAGCAGAGC

                                                                                                                 235 BamHI
                                                                                                        227 KpnI
TCGTTTAGTGAACCGTCAGATCGCCTGGAGACGCCATCCACGCTGTTTTGACCTCCATAGAAGACACCGGGACCGATCCAGCCTCCGCGGCCCCGAATTCGAGCTCGGTACCCGGGATCC
ACCGGCCGGTCGCCACCATGGTGAGCAAGGGCGAGGAGCTGTTCACCGGGGTGGTGCCCATCCTGGTCGAGCTGGACGGCGACGTAAACGGCCACAAGTTCAGCGTGTCCGGCGAGGGCG
AGGGCGATGCCACCTACGGCAAGCTGACCCTGAAGTTCATCTGCACCACCGGCAAGCTGCCCGTGCCCTGGCCCACCCTCGTGACCACCCTGACCTACGGCGTGCAGTGCTTCAGCCGCT
ACCCCGACCACATGAAGCAGCACGACTTCTTCAAGTCCGCCATGCCCGAAGGCTACGTCCAGGAGCGCACCATCTTCTTCAAGGACGACGGCAACTACAAGACCCGCGCCGAGGTGAAGT
TCGAGGGCGACACCCTGGTGAACCGCATCGAGCTGAAGGGCATCGACTTCAAGGAGGACGGCAACATCCTGGGGCACAAGCTGGAGTACAACTACAACAGCCACAACGTCTATATCATGG
CCGACAAGCAGAAGAACGGCATCAAGGTGAACTTCAAGATCCGCCACAACATCGAGGACGGCAGCGTGCAGCTCGCCGACCACTACCAGCAGAACACCCCCATCGGCGACGGCCCCGTGC
TGCTGCCCGACAACCACTACCTGAGCACCCAGTCCGCCCTGAGCAAAGACCCCAACGAGAAGCGCGATCACATGGTCCTGCTGGAGTTCGTGACCGCCGCCGGGATCACTCTCGGCATGG
ACGAGCTGTACAAGTAAAGCGGCCGCGACTCTAGATCATAATCAGCCATACCACATTTGTAGAGGTTTTACTTGCTTTAAAAAACCTCCCACACCTCCCCCTGAACCTGAAACATAAAAT
```

Underline, CMV minimal promoter
Green, EGFP coding sequence

Fig. S6. Sequences of the *X. laevis prph* promoter, intron 1, primers, and vectors used for construct generation

Biology Open • Supplementary information
